# Influence of early elective tracheostomy on the incidence of postoperative complications in patients undergoing head and neck surgery

**DOI:** 10.1186/s12871-019-0715-9

**Published:** 2019-03-28

**Authors:** Johannes Meier, Michael Wunschel, Anne Angermann, Tobias Ettl, Thomas Metterlein, Christoph Klingelhöffer, Torsten E. Reichert, Markus Ritzka

**Affiliations:** 1 Department of Oral and Maxillofacial Surgery of the University Medical Center Regensburg, Franz-Josef-Strauss-Allee 11, 93053 Regensburg, Germany; 2 Department of Anesthesiology of the University Medical Center Regensburg, Franz-Josef-Strauss-Allee 11, 93053 Regensburg, Germany

**Keywords:** Airway, Head and neck surgery, Tracheostomy, Delirium, Pneumonia

## Abstract

**Background:**

The incidence of postoperative complications after head and neck surgery is high. This study evaluated the influence of early elective tracheostomy on the incidence of postoperative pneumonia and delirium.

**Methods:**

We reviewed the data of all patients who had undergone removal of an oropharyngeal tumor and microsurgical tissue transfer at our department in a two year period. Pearson’s Chi-squared test and the Fischer’s exact t-test were then used to measure the influence of patients’ preexisting conditions and risk factors and of early elective tracheostomy on the incidence of postoperative complications.

**Results:**

In total, 47 cases were analyzed. Patients with an endotracheal tube were ventilated for a longer time (3.4 days vs. 1.5 days) and were transferred to the regular ward later (after 6.9 days vs. 4.7 days) than patients with tracheostomy. Only 1 (2.1%) of the patients with a tracheostomy developed pneumonia in contrast to 5 intubated patients (10.6%) and only 2 patients with a tracheostomy developed postoperative delirium (9.5%) in contrast to 8 intubated patients (30.8%).

**Conclusion:**

Early primary tracheostomy in patients undergoing resection of oropharyngeal cancer seems to have numerous benefits, such as lower complication rates with regard to pneumonia and postoperative delirium and shorter duration of both mechanical ventilation and intensive care unit (ICU) stays. Further studies have to evaluate if these benefits also influence morbidity and mortality rates.

## Background

Every year, over 500.000 new cases of head and neck squamous cell carcinoma are diagnosed worldwide [1]. Oral and pharyngeal cancer, grouped together, represent the sixth most common tumor entity [2]. Several national and international guidelines are available on how to choose the best options for diagnosis and treatment [3, 4]. Currently, the first-line treatment in most cases is surgical removal. Other therapeutic options are emerging, but right now their use is limited to special cases or study conditions [5]. Standard treatment includes complete removal of the tumor mass with a sufficient safety margin, generally in combination with some form of neck dissection according to the initial staging. Immediate reconstruction with a microvascular transplant is desired if applicable [3]. Overall perioperative complication rates are up to 50% [6]. Because of the extent and complexity of these surgical interventions, special attention must also be drawn to the airway. Primary elective tracheostomy is a well-established means of airway management in head and neck surgery, although no precise indication criteria are available so far [7].

The incidence of respiratory complications, particularly pneumonia, after head and neck surgery is high, ranging from 10 to 47% [8]. Another important complication is postoperative delirium [9]. Reviews have suggested that at least 25% of patients undergoing head and neck surgery are affected by postoperative delirium. However, this percentage could underestimate the risk [10].

In our study, we evaluated if early elective tracheostomy influences the incidence of postoperative pneumonia and delirium by means of a retrospective data analysis.

## Methods

We reviewed all records of patients who had undergone removal of an oropharyngeal tumor and microsurgical tissue transfer at the Department of Oral and Maxillofacial Surgery of the University Medical Center Regensburg in a defined period of time of two years (2009 and 2010). During this period medical care and operations were performed by a consistent and experienced medical and surgical team. This period was set in order to try to exclude confounding factors like a learning curve. Data were collected from the medical records of in-patients and from the program Metavision® used for documentation on intensive care. Only data of in-patient treatments were analyzed.

The following patient-related parameters were compiled: age, sex, past medical history, substance abuse, UICC tumor stage and grade, and American Society of Anesthesiologists (ASA) physical score (for details see Table 1).

**Table 1:** case overview

ID	age	flap	stage	ASA	nicotine	C2	airway	delirium	pneumonia	duration of ventilation [dd:hh:mm]	stay on ICU [d]
1	74	pf	4	2	1	1	sec. TS	1	0	10:04:35	13
2	52	mvf	4	2	1	1	intubated	0	0	0:21:00	3
3	72	pf	2	3	0	0	intubated	0	0	0:19:20	3
4	55	mvf	3	1	0	0	prim. TS	0	0	2:15:37	4
5	74	mvf	1	3	0	0	intubated	1	0	1:21:08	4
6	66	pf	1	3	0	0	prim. TS	0	0	0:00:00	2
6.1	67	mvf		3	0	0	prim. TS	0	0	1:15:29	3
7	72	mvf	1	3	0	0	prim. TS	0	0	1:00:54	3
8	79	pf	4	3	0	0	intubated	1	0	0:16:48	5
9	81	pf	2	3	0	0	extubated	0	0	0:00:00	2
10	54	mvf	2	3	0	0	intubated	0	0	1:16:50	4
11	48	mvf	4	2	1	1	intubated	1	0	2:15:50	6
12	51	mvf	4	2	1	1	prim. TS	0	0	2:19:00	5
13	81	mvf	4	3	0	0	prim. TS	1	0	1:17:45	8
14	49	mvf	3	3	0	0	intubated	0	0	1:21:33	4
15	53	mvf	2	2	1	0	intubated	0	0	1:19:42	5
16	50	mvf	3	2	1	0	sec. TS	0	0	3:15:57	9
17	47	mvf	4	3	1	1	sec. TS	1	0	4:22:03	10
18	59	pf	2	3	1	1	sec. TS	0	1	0:17:57	5
19	67	mvf	4	2	0	1	prim. TS	0	0	0:12:00	3
20	43	mvf	4	2	0	1	prim. TS	0	0	0:14:21	3
21	78	pf	4	3	0	0	extubated	0	0	0:00:00	2
22	63	mvf	3	3	1	1	intubated	0	0	1:00:43	3
23	64	pf	4	3	0	1	intubated	1	1	8:20:45	15
24	78	mvf	4	2	0	0	intubated	0	0	2:19:39	5
25	71	mvf	4	3	0	0	prim. TS	0	0	1:17:00	4
26	46	mvf	4	3	0	1	prim. TS	0	0	0:00:00	3
27	75	pf	4	3	0	0	prim. TS	0	0	0:12:28	3
28	71	mvf	1	2	0	0	intubated	0	0	2:15:21	5
28.1	71	mvf	2	3	0	0	intubated	0	1	13:12:54	26
29	52	mvf	4	3	1	0	prim. TS	0	0	0:03:00	2
30	85	mvf	2	2	1	0	prim. TS	0	1	7:00:00	27
31	67	mvf	4	3	1	1	intubated	1	1	9:16:24	19
32	38	mvf	4	3	1	1	intubated	1	1	8:14:45	10
33	47	mvf	3	2	0	0	prim. TS	0	0	0:04:32	2
34	52	pf	3	3	1	1	prim. TS	0	0	0:10:17	3
35	53	mvf	3	2	1	0	prim. TS	0	0	0:23:32	3
36	53	pf	2	2	1	1	prim. TS	1	0	0:12:45	4
37	65	mvf	4	3	1	1	prim. TS	0	0	0:20:20	2
38	76	mvf	4	2	0	0	intubated	0	0	0:20:51	3
39	57	mvf	3	2	0	0	intubated	0	0	2:01:29	4
40	68	pf	3	3	1	1	intubated	0	0	0:21:30	3
41	66	pf	3	2	1	1	intubated	0	0	0:03:18	2
42	51	mvf	4	2	1	0	prim. TS	0	0	1:14:30	5
43	57	mvf	1	2	1	1	prim. TS	0	0	1:00:00	3
44	52	mvf	3	2	0	0	prim. TS	0	0	4:02:34	7
44.1	53	mvf		3	0	0	intubated	0	0	4:19:13	8

Perioperative treatment: the type of anesthesia and postoperative airway management (extubation, tracheostomy, and endotracheal tube). Tracheostomy that was indicated preoperatively or during the course of the operation was regarded as primary or early elective tracheostomy. The procedure was performed as an open surgical tracheostomy as described in various sources [11, 12]. Tracheostomy performed in the postoperative course due to medical problems leading to prolonged ventilation was classified as secondary tracheostomy.

Postoperative course: postoperative sedation according to the Richmond Agitation-Sedation Scale (RASS), postoperative pain level on a numeric rating scale (NRS), postoperative ventilation, postoperative delirium, postoperative complications (transplant-associated and medical) as well as the type and timing of revision surgery. Screening for postoperative delirium was done by applying the Intensive Care Delirium Screening Checklist (ICDSC) once every eight-hour shift and diagnosis of postoperative delirium was made with a score of equal to or more than 4 [13]. To establish the diagnosis of ventilator associated pneumonia (VAP) several clinical (temperature, oxygenation status), laboratory (leukocytes, C-reactive protein (CRP), procalcitonin (PCT)) and radiologic findings (chest radiograph or CT) were used [14]. A course of intravenous Piperacillin/Tazobactam was then started and specimens obtained by bronchoalveolar lavage (BAL) were sent away for microbiological analysis and resistance testing. Antibiotic therapy was then modified according to laboratory findings.

### Data analysis

All data were anonymized and summarized in charts generated with MS Excel (Microsoft, Redmond, USA) and analyzed by a professional statistician using IBM SPSS Statistics 20.0 (IBM, Armonk, USA). The Pearson’s Chi-squared test and the Fischer’s exact t-test were used for statistical analysis. *P*-values of < 0.05 were regarded as statistically significant.

## Results

In the observation period, 44 patients with the diagnosis of oropharyngeal carcinoma had undergone surgery at the University Medical Center Regensburg. 5 (10.6%) patients were diagnosed with UICC stage I cancer, 8 (17%) patients with stage II, 11 (23.4%) patients with stage III and 21 (44.7%) with stage IVa. Upon consultation with the institutional tumor board, all patients underwent complete (R0) tumor resection and reconstruction, either with a microvascular anastomosed free flap (mvf) or a pedicled regional flap (pf). In total, 47 flap transfers were performed. Three patients received a second flap transfer 5, 8 and 13 months respectively after their first operation and were included into analysis as separate cases but marked by assigning the same case number “.1”. The procedure and the peri- and postoperative course followed institutional standards (find an excerpt of the data gathered in Table 1).

In brief, all patients received balanced anesthesia with sufentanil and sevoflurane. To maintain adequate perfusion pressure, proper volume status was maintained. Hypotension was treated with vasopressors. Postoperative sedation was maintained with intravenous propofol 2% (postoperative dosages from 0 to 800, mean 236 + − 153 mg/h) and sufentanil for 12 h after surgery to avoid acute complications such as graft thrombosis or bleeding. After discontinuation of sedation and with of adequate spontaneous breathing, ventilation was discontinued. Patients with an endotracheal tube were extubated when airway patency was given. Cardiopulmonary stable patients were transferred to the regular ward the next day.

Thirty-three of the 47 tissue transfer patients were male, and 14 female, which resulted in a ratio of f:m of 1:2.4. At the time of surgery, mean age was 62 years; the youngest patient was 38 years and the oldest 85 years of age. Mean age of the female patients was 59 years, that of the male patients 63 years. Patients were also grouped in two groups according to an age of 70 or older (15 patients) and under 70 years (32 patients) in order to explore the influence of age on the postoperative outcome.

Nineteen (40.4%) of the patients had a history of alcohol abuse and 21 (44.7%) did smoke. 1 (2.1%) patient was classified as ASA 1 whereas 20 (42.6%) and 26 (55.3%) were grouped into ASA classes 2 and 3 respectively.

Two patients had been primarily extubated in the operation theater.

Patients with an endotracheal tube were ventilated for 3.4 (+ − 3.73) days, whereas patients with tracheostomy could be weaned significantly earlier (1.5 + − 1.66 days, *p* = 0.005). Patients who were primarily intubated were transferred to the regular ward after 6.9 + − 6.2 days, whereas patients with a tracheostomy could be transferred after 4.9 + − 5.4 days (*p* = 0.266).

Only 1 (2.1%) of the patients with a tracheostomy developed pneumonia in contrast to 5 intubated patients (10.6%, *p* = 0.139). Patients with pneumonia had to be ventilated for 8.1 + − 4.2 days and were discharged from intensive care unit (ICU) after 17 + − 8.7 days in contrast to patients without pneumonia (1.7 + − 1.9 days ventilation, *p* = 0.032, 4.3 + − 2.4 days ICU, *p* < 0.001).

Two patients who had undergone primary elective tracheostomy developed postoperative delirium (9.5%) in contrast to 8 intubated patients (30.8%, *p* = 0.078). Two of these patients received a secondary tracheostomy because of a prolonged need for mechanical ventilation.

In total, 4 patients underwent secondary tracheostomy 5 to 8 days after the initial tumor surgery due to severe airway swelling.

When patients were analyzed in different subsets according to age groups, 1 of the 5 patients of an age of 70 or older with primary tracheostomy developed postoperative delirium while 3 of the 10 primarily intubated patients experienced this complication.

Neither the ASA physical score, initial dosages of sedatives nor a history of nicotine or alcohol abuse, or age, even when analyze in subgroups as specified above, had any influence on the duration of ventilation, the rates of pneumonia or delirium, or the time point of ICU discharge.

## Discussion

Resection of oropharyngeal cancer is a complex procedure that often requires different flaps for covering the tissue defect. The airway is of special importance because local complications may lead to catastrophic consequences. So far, the question whether primary tracheostomy is more beneficial for patients than prolonged endotracheal intubation has hardly been investigated in prospective studies.

This study compared the influence of the airway on immediate postoperative complications such as pneumonia or delirium. It was shown that patients with a primary tracheostomy required a shorter sedation and ventilation. Since sedation itself is a risk factor for developing both pneumonia and delirium, it is understandable that tracheostomized patients experience fewer complications. Another recently published study yielded similar results. The duration of ventilation was longer in non-tracheostomized patients than in tracheostomized patients [7]. In our institution as it is in most units caring for critical ill patients prevention of (postoperative) delirium is seen as a very important part of intensive care therapy. We implemented delirium prevention as a standard operation procedure (SOP) using a multimodal approach in management of pain, agitation and delirium (PAD) [15, 16]. Aims are a pain level of NRS < = 3, sedation on a level of − 1 to 0 on RASS and ICDSC > = 4. Measures to reach these goals are, besides sufficient pain medication, mainly early mobilization and physical activation which can be performed much more easily with patients that are independent of a mechanical respirator. We do not use pharmacologic medication for delirium as a matter of routine. Treatment of postoperative delirium consists of pharmacological and non-pharmacological therapy. Pharmacological therapy uses alpha-2-agonists like Clonidine, anti-psychotic drugs, non-benzodiazepine sedation like Propofol and benzodiazepines in selected cases [16]. Non-pharmacological therapy concentrates on cognitive stimulation and creating a familiar environment [16]. These measures are also facilitated by reducing the length of stay on ICU. Another important risk factor for developing postoperative delirium is advanced age [17]. Several factors like neuronal aging, neuroinflammation, neurotransmitter deficiency, neuroendocrine activation and brain network connectivity change have been identified as causative in postoperative delirium [18]. These factors have been associated with growing age and it is believed that they contribute to an increased vulnerability and a lack of physiologic reserve in the elderly [18]. In our study, age of the patients did not influence the probability of the occurrence of postoperative delirium. The reason for this different finding lies most likely in the low number of cases in the group of patients with high age.

Another important postoperative complication was the development of nosocomial pneumonia or VAP. 12.8% of all patients showed corresponding symptoms, which prolonged their duration of ventilation and their stay on intensive care. This rate lies within the range published elsewhere [19, 20]. All patients had shown first symptoms of pneumonia within 4 days of their stay on intensive care, which per definition classifies as ‘early-onset’ nosocomial pneumonia [21]. In most cases Serratia marcescens, Enterococci and *Enterobacter cloacae* were identified as causative agents. Klebsiellae, coagulase-nagative Staphylococci, *Escherichia coli* and *Proteus mirabilis* were found in single cases. This spectrum of germs differs a bit from that given in literature, where Enterobacteriaceae, *Staphylococcus aureus*, *Pseudomonas aeruginosa* and Acinetobacter baumannii have been isolated in most cases of nosocomial pneumonia [21]. Of the 6 patients with nosocomial pneumonia, 5 were ventilated through a nasal tube and only 1 through tracheostomy. Because of the low number of patients, this difference was not significant but may indicate another advantage of early tracheostomy. This finding contradicts results reported by other groups, where tracheostomy was found to be an independent predictor of medical complications [7, 22, 23]. Especially a naso-endotracheal tube can function as guide for bacteria located in the sinuses and oral cavity compared to the tracheostomy tube. From a practical point of view oral hygiene and bronchial suction is easier to perform after tracheostomy than in the presence of an oropharyngeal tube. Weaning can be performed quicker since transition between controlled and spontaneous breathing is easier.

Another finding of this study was that patients with primary tracheostomy were discharged earlier from the ICU. This fact and the lower complication rate are of interest, particularly from an economic point of view. Both complication management and ICU treatment take up valuable resources. Because ICU stays include the risk of higher complication rates, minimizing ICU treatment is of corresponding value.

Initially, the time for postoperative sedation and monitoring in intensive care was set to 12 h after surgery. The average length of stay in the intensive care unit was 5.9 days with a median of 4.0 days. This period was considerably longer than intended and also what has been described in the literature [7, 8]. Reasons for the delayed discharge were mainly flap-associated complications such as swelling or thrombosis, which increased the duration of ventilation and subsequently the risk of developing medical complications such as pneumonia or delirium.

However, it should be noted that tracheostomy as a surgical procedure itself carries the risk of complications [22]; literature reports have described a complication rate of 8% with 72% of complications occurring in the immediate or early postoperative phase [24]. Our patients, however, had not developed any tracheostomy-related complications. Given this different findings, selecting the right patient for elective tracheostomy seems to be the key for improving clinical results.

## Conclusion

Early primary tracheostomy in patients undergoing resection of oropharyngeal cancer seems to have numerous benefits, such as lower complication rates with regard to pneumonia and postoperative delirium and shorter duration of both mechanical ventilation and ICU stays. Further studies have to evaluate if these benefits also influence morbidity and mortality rates.

### Aknowledgements

We would like to thank Mrs. Monika Schoell for her assistance with the study.

## Abbreviations

ASA: American Society of Anesthesiologists
BAL: Bronchoalveolar lavage
CRP: C-reactive protein
ICDSC: Intensive Care Delirium Screening Checklist
ICU: Intensive care unit
mvf: microvascular anastomosed free flap
NRS: Numeric rating scale
PAD: Pain, agitation and delirium
PCT: Procalcitonin
pf: pedicled regional flap
prim. TS: primary elective tracheostomy
RASS: Richmond Agitation-Sedation Scale
sec. TS: Secondary tracheostomy
SOP: Standard operation procedure
VAP: ventilator associated pneumonia
